# An anastomosing hemangioma mimicking a renal cell carcinoma in a kidney transplant recipient: a case report

**DOI:** 10.1186/s12882-021-02467-y

**Published:** 2021-07-13

**Authors:** Chang Seong Kim, Soo Jin Na Choi, Sung-Sun Kim, Sang Heon Suh, Eun Hui Bae, Seong Kwon Ma, Soo Wan Kim

**Affiliations:** 1 Department of Internal Medicine, Chonnam National University Medical School, 160 Baekseo-ro, Dong-gu, Gwangju, 61469 South Korea; 2 Department of Surgery, Chonnam National University Medical School, Gwangju, South Korea; 3 Department of Pathology, Chonnam National University Medical School, Gwangju, South Korea

**Keywords:** Anastomosing hemangioma, Mass, Kidney transplantation, Case report

## Abstract

**Background:**

Although anastomosing hemangiomas are very rare and benign vascular neoplasms, these tumors are more common among patients with end-stage kidney disease. Incidental finding of these tumors in the kidney or adrenal gland has been reported. Herein, we describe a case in which an anastomosing hemangioma was misdiagnosed as a renal cell carcinoma before kidney transplant.

**Case presentation:**

A 35-year-old woman with lupus nephritis was admitted to our emergency department for suspected uremic symptoms of nausea and general weakness. She had received hemodialysis due to end-stage kidney disease, and a living-donor kidney transplantation from her father was planned. On pre-operative contrast-enhanced computed tomography and magnetic resonance imaging, a 1.7 cm renal cell carcinoma was observed in the right kidney. On staining after radical nephrectomy, irregularly shaped vascular spaces of various sizes were observed, with these spaces having an anastomosing pattern. As the findings of the anastomosing hemangioma are similar to those of a renal cell carcinoma on imaging, histology examination was necessary to confirm the diagnosis of anastomosing hemangioma and to prevent delay in listing for kidney transplantation. Good kidney function was achieved after transplantation, with no tumor recurrence.

**Conclusion:**

Our case underlines the importance for prompt surgical resection of an enhancing renal mass to confirm diagnosis in patients scheduled for kidney transplantation to avoid any delay.

## Background

Renal cell carcinoma is the most common subtype of kidney cancer in patients with end-stage kidney disease (ESKD), although vascular kidney tumors are a rare occurrence [1]. Anastomosing hemangiomas develop frequently in patients with ESKD [2]. Although an anastomosing hemangioma is a benign vascular tumor, the radiological imaging findings of these tumors are similar to those of renal cell carcinomas [3, 4].

Previous studies have reported on the incidental detection of anastomosing hemangiomas in the kidneys or adrenal glands [3]. To the best of our knowledge, however, the misdiagnosis of an anastomosing hemangioma as a renal cell carcinoma during the medical work-up before kidney transplantation has not been previously reported. Herein, we report a case of anastomosing hemangioma confirmed by histological examination after nephrectomy, which prevented a delay in the waiting period to living-donor kidney transplantation.

## Case presentation

A 35-year-old woman with a history of hypertension, severe osteoporosis, and stage 5 chronic kidney disease due to lupus nephritis, was admitted to our emergency department for suspected uremic symptoms of nausea and general weakness. Her vital signs were normal, with a heart rate of 80 beats/min and blood pressure of 100/60 mmHg. However, her blood urea nitrogen (179.4 (reference range: 8–23) mg/dL) and serum creatinine (10.9 (reference range: 0.5–1.3) mg/dL) levels were markedly increased, with her serum inorganic phosphate level also higher than normal at 8.4 (reference range: 2.5–5.5) mg/dL. The patient was treated with emergent hemodialysis and a living-donor kidney transplantation, from her father, was planned.

**Fig. 1:**
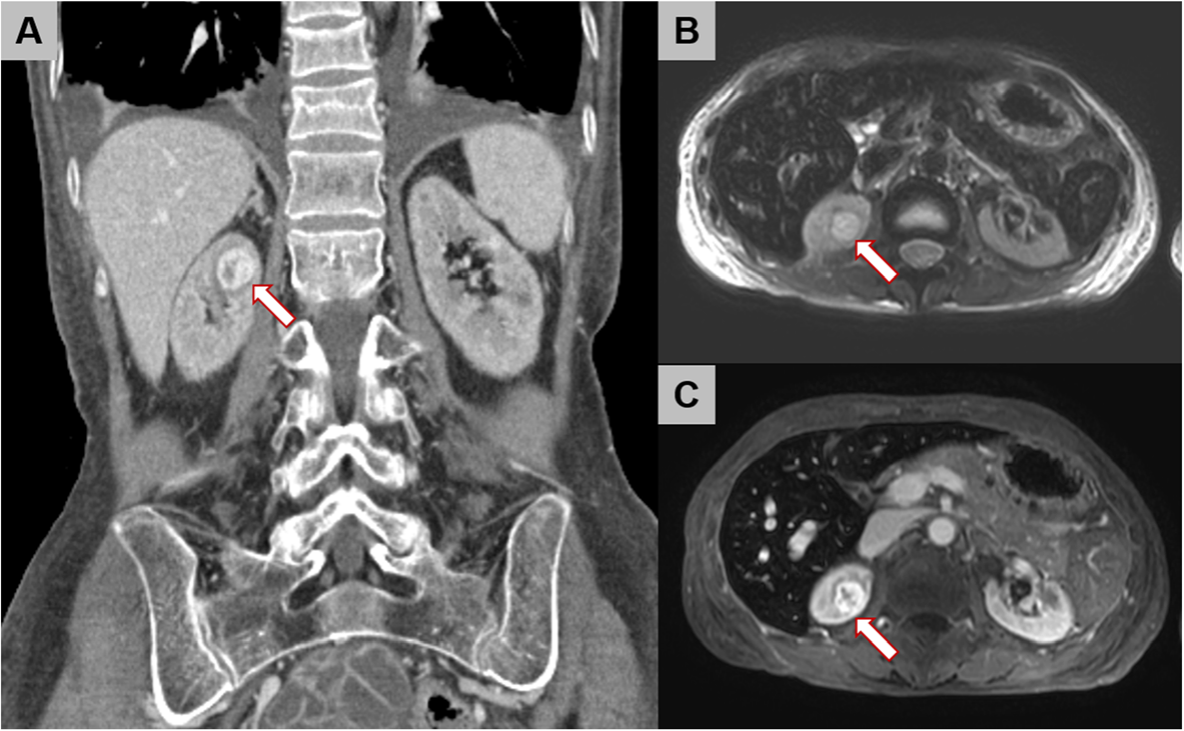
**A** Contrast-enhanced computed tomography scan revealed the heterogeneously enhancing mass in the right kidney (arrow). Kidney magnetic resonance imaging revealed the **B** lesion was a high signal intensity in T2-weighted image, and **C** showed heterogeneous enhancement (arrow)

**Fig. 2:**
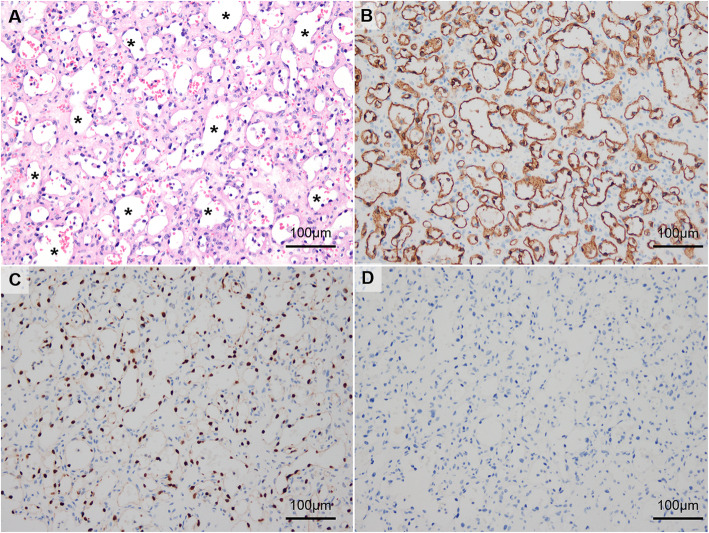
**A** Hematoxylin and eosin stain showed irregular shaped angiomatous spaces, which are lined by single-layered endothelial cells with occasional hobnail feature (asterisks). These endothelial cells are immunopositive for **B** CD34 and **C** ETS-related gene, while not for **D** podoplanin. Images were acquired using an upright microscope and microscope digital camera (BX43 and DP73; Olympus, Tokyo, Japan). Original magnification × 200

During the pre-transplantation medical work-up, contrast-enhanced computed tomography (CT) of the abdomen revealed a heterogeneously enhancing mass, 1.7 cm in diameter, located in the upper pole of the right kidney (Fig. 1A). On follow-up magnetic resonance (MR) imaging, the mass presented high signal intensity on T2-weighted images, with heterogeneous enhancement in the right kidney (Fig. 1B and C). Based on findings of the MR imaging, a diagnosis of renal cell carcinoma, stage T1aN0, was made. As the right renal mass was small, with no associated symptoms, simultaneous right radical nephrectomy and kidney transplant was planned by the transplant surgeon and urologist. Open radical nephrectomy was performed via a subcostal incision; the patient was then positioned for the kidney transplant. Hematoxylin and eosin staining performed after nephrectomy revealed irregularly shaped vascular spaces of various sizes, with an anastomosing pattern (Fig. 2A). On immunostaining, the sample was positive for CD34, CD31, and ETS-related genes and for friend leukemia integration 1 transcription factor and negative for podoplanin, human herpes virus-8, and glucose transporter-1 on immunostaining (Fig. 2B–D). Based on these findings, a final diagnosis of anastomosing hemangioma was made. After kidney transplantation, good renal function was achieved, with no tumor recurrence.

## Discussion and conclusions

In 2009, Montgomery and Epstein first described an anastomosing hemangioma of the genitourinary tract, with the conclusion that such hemangiomas were rare and benign in contrast to angiosarcomas [5]. Therefore, nephrectomy is not clinically required for this benign vascular neoplasm. As well, patients with ESKD who have an anastomosing hemangioma can be immediately listed for living-donor kidney transplantation or registered for deceased-donor kidney transplantation. The difficulty, however, is that the imaging findings for anastomosing hemangiomas are similar to those for renal cell carcinomas, including heterogeneous enhancement of lesions on CT and hyperintensity on T2-weighted MR images [6]. As subcutaneous biopsy of vascular lesions may pose a challenge owing to the risk of profound bleeding [3], anastomosing hemangiomas have been diagnosed by nephrectomy in the majority of reported cases, including ours.

There is controversy regarding the management of incidentally diagnosed kidney cancer during the medical work-up for kidney transplantation [7–13]. The most worrisome for these patients is an unnecessary delay of the kidney transplant. As such, concurrent radical nephrectomy and kidney transplant is recommended for these patients. Of note, partial nephrectomy is recommended for small solitary renal cell carcinomas because of the positive nephron-sparing effect [14]. According to a recent review, the median size of kidney anastomosing hemangiomas is 1.5 (range, 0.1–8.0) cm, with the hemangioma being < 4 cm in the majority of cases [15]. According to current guidelines, radical nephrectomy would be recommended for these patients for diagnosis or treatment. We adhered to these guidelines in our case, proceeding with right radical nephrectomy, although the tumor was small, with a diameter of 1.7 cm. Partial nephrectomy may have been suitable to preserve residual renal function; however, her residual renal function had already decreased, consistent with ESKD, and, thus, radical nephrectomy was warranted to secure adequate surgical safety margins of the tumor.

In conclusion, the principal finding of our case was the misdiagnosis of an anastomosing hemangioma as a renal cell carcinoma based on CT and MR imaging in a patient with ESKD. As a living transplant was planned for our patient, we proceeded with prompt surgical resection of the heterogeneous enhancing renal mass to avoid delay in the transplant.

## Data Availability

All the relevant data and materials of our patient are present in the manuscript but in case the original copy of the documents are needed, they are available from the corresponding author on reasonable request.

## Abbreviations

CT: Computed tomography
MR: Magnetic resonance
ESKD: End-stage kidney disease
